# The effect of surface roughness on the Er:YAG laser-induced photoacoustic removal of bacteria in zero-gap periodontal/*peri*-implant pocket model^☆^

**DOI:** 10.1016/j.ultsonch.2025.107458

**Published:** 2025-07-07

**Authors:** Dominik Šavli, Marko Volk, Katja Molan, Saša Terlep, Špela Levičnik-Höfferle, Aleš Babnik, Mojca Trost, Boris Gašpirc, Matjaž Lukač, David Stopar, Matija Jezeršek

**Affiliations:** a University of Ljubljana, Faculty of Mechanical Engineering, Aškerčeva Cesta 6, 1000 Ljubljana, Slovenia; b University of Ljubljana, Biotechnical Faculty, Department of Microbiology, Večna Pot 111, 1000 Ljubljana, Slovenia; c Fotona d.o.o., Stegne 7, 1000 Ljubljana, Slovenia; d University of Ljubljana, Medical Faculty, Department of Oral Medicine and Periodontology, Vrazov Trg 2, 1000 Ljubljana, Slovenia; e Institut Jozef Stefan, Jamova 39, 1000 Ljubljana, Slovenia; f University of Ljubljana, Faculty of Mathematics and Physics, Jadranska 19, 1000 Ljubljana, Slovenia; g University of Novo Mesto, Faculty of Health Sciences, Na Loko 2, 8000 Novo mesto, Slovenia

**Keywords:** Er:YAG laser, Cavitation dynamics, Biofilm removal, Surface roughness, Photoacoustic cleaning, Periodontal and *peri*-implant pockets, Secondary cavitation

## Abstract

Effective biofilm removal from periodontal and *peri*-implant pockets remains a challenge due to constrained geometry and limited access. This study investigates a novel phenomenon of distant-field cleaning utilizing Er:YAG laser treatment, where removal of bacteria occurs in areas without previously observed cavitation under high-speed imaging. To understand this effect, we developed a transparent zero-gap model simulating a tooth or implant and surrounding soft tissue. We systematically examined the impact of laser fiber insertion depth, cavitation bubble dynamics, the stiffness and roughness of the material, and laser parameters on the cleaning efficiency.

Our findings reveal that the removal of bacteria indeed correlates strongly with cavitation occurrence. Deeper optical fiber insertion into the pocket model only enhanced cleaning efficiency by moving the fluid dynamics and enabling deeper water penetration. Surprisingly, high-speed imaging showed no cavitation in distant regions, raising questions about the mechanisms enabling such cleaning. Further investigation uncovered that surface roughness played a critical role in facilitating this distant-field effect. The smooth, transparent surfaces used in imaging experiments suppressed fluid dynamics, while textured surfaces created by 3D-printed molds and bacterial monolayer allowed deeper water penetration and pressure wave propagation. These surface irregularities enabled localized cavitation events and enhanced bacterial disruption, even in regions beyond the laser fiber’s immediate influence.

This study emphasizes the significance of surface roughness in test models, highlighting the need for models to closely mimic the conditions of real clinical scenarios for accurate optimization of Er:YAG laser-induced photoacoustic removal of bacteria.

## 1 Introduction

Biofilm removal from periodontal and *peri*-implant pockets remains a critical challenge in modern dentistry. Especially problematic is biofilm removal from narrow apical regions since they are inaccessible to mechanical instruments. The bacterial colonization and the formation of biofilms are key etiological factors for development of periodontal and *peri*-implant diseases [1,2].

Over the past decade, several techniques have been explored to remove bacterial deposits within periodontal and *peri*-implant pockets, each varying in invasiveness and effectiveness. Techniques based on atorvastatin gel [3] or nanoparticles [4] have shown promise but face limitations in effectiveness in difficult-to-reach areas. Ultrasonic irrigation typically requires direct contact between the tip and the target tissue, limiting the formation of cavitation in areas distant from the tip. For optimal effectiveness, the tip must operate within a space at least three times its diameter, resulting in reduced efficiency of the technique in mechanically hard-to-reach areas.

In contrast, Er:YAG laser-induced cavitation occurs in distant areas from the laser tip, allowing effective cleaning of hard-to-reach areas [5,6]. This, so-called laser-induced photoacoustic cleaning, especially by using Er:YAG lasers, has demonstrated promising non-invasive capabilities in biofilm disruption through photoacoustic cleaning mechanisms even in difficult-to-reach areas [7]. The fundamental mechanism of laser-induced photoacoustic cleaning is based on high absorption of Er:YAG laser light (2940 nm) delivered through the fiber tip (FT) in water leading to the generation of a rapidly expanding vapor bubble [8]. This creates significant pressure fluctuations, which in turn disrupts the biofilm structure by inducing mechanical stress and fluid dynamics. This dynamic process extends well beyond the immediate vicinity of the vapor bubble. Unlike mechanical methods, laser-induced cavitation and pressure waves can reach regions beyond possible physical contact with minimal collateral effects [9,10,11].

In this study, the term laser-induced photoacoustic cleaning refers to the above explained mechanism. This usage differs from photoacoustic imaging techniques, where laser-induced acoustic signals are detected for diagnostic purposes.

Recent advancements in ultrafast imaging have enhanced our understanding of these dynamics, with studies employing megahertz X-ray imaging to resolve transient cavitation events in fluid–solid interactions [12,13]. For instance, Eskin et al. demonstrated that X-ray free electron laser imaging can capture ultrasound-induced cavitation during 2D material exfoliation, revealing the role of pressure waves and microbubble dynamics in confined geometries [13].

Previous studies employing wedge geometry have demonstrated that primary cavitation bubbles (PB) consistently form at the FT, which in turn reliably trigger secondary cavitation (SC) at distant and difficult to reach locations within the wedge [10,11]. Despite the clear demonstration of cavitation's effectiveness in these model systems, it is important to note that such wedge geometries are less common in clinical practice compared to situations where the FT is tightly confined between the tooth and gingival tissue.

To address this discrepancy and enhance the clinical relevance, a zero-gap model system simulating periodontal/*peri*-implant pocket has been introduced. This model closely mimics these clinically relevant conditions and enables a more detailed investigation of biofilm disruption mechanisms from extremely narrow and difficult-to-access areas within periodontal and *peri*-implant pockets [14]. However, in such narrow spaces, surface roughness becomes an important parameter affecting wettability [15,16], fluid dynamics [17], and cavitation threshold [18], yet it has not been previously considered.

Previous research on laser-induced photoacoustic cleaning of dental root canals [19,20,21] and simulated periodontal/*peri*-implant pockets [10,22] has shown that employing the dual-pulse Auto-SWEEPS (ASW) laser modality marked a significant advancement. The ASW modality incrementally in 10 µs steps varies the delay between the two pulses, from 200 to 650 µs [19], ensuring that at some point during each cycle, the delay aligns optimally to maximize the generation of SC enhancing cleaning efficiency.

Building on our previous work, this study advances the understanding of laser-induced photoacoustic cleaning within zero-gap model system, particularly focusing on the impact of surface roughness in zero-gap model systems. It examines various parameters like laser modality and energy, FT depth, dual-pulse cavitation dynamics, and material properties in relation to efficiency of disrupting bacteria. The research experimentally investigates removal of bacteria and cavitation development, detailing how parameters affect the cleaning process while highlighting a critical, previously underexplored impact of surface texture within zero-gap model on cleaning efficacy on various distances from the FT. These findings highlight the importance of realistic experimental models that mimic the complexities of clinical conditions.

## 2 Materials and methods

**Fig. 1:**
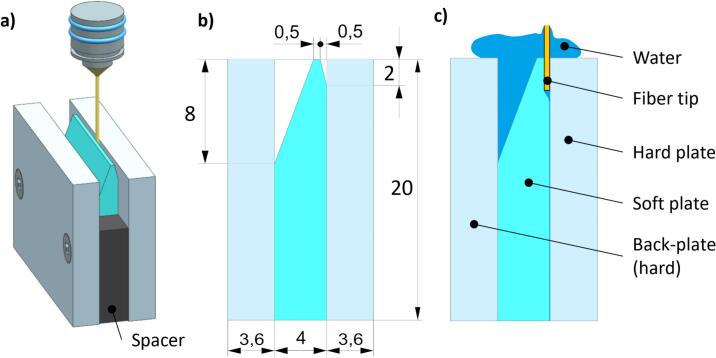
Zero-gap model configuration for experiment of bacteria removal with Er:YAG photoacoustic laser treatment. a) 3D presentation with all parts of the model shown. b) Initial setup with dimensions marked before FT insertion. c) Model with the fiber tip and rinsing water in place.

Experiments were conducted in-vitro utilizing a model system designed to simulate a periodontal/*peri*-implant pocket, as shown in Fig. 1. The model consists of two basic components, soft plate made of polydimethylsiloxane (PDMS) and hard glass-made plate, simulating hard tissue/implant, respectively.

The hard glass plate has Young’s modulus E ≈ 70 GPa, which is close to the enamel E ≈ 80 GPa [23], while the stiffness of PDMS used in the model was varied by adjusting the cross-linker concentration, yielding storage moduli (G') of 14–29 kPa, corresponding to E ≈ 42–87 kPa (assuming E ≈ 3G' for elastomers [24]). The consistency and stiffness of PDMS plates were further validated by a panel of seven dentists who manually assessed the tactile similarity of the PDMS samples to human gingival tissue during simulated periodontal probing [10].

In addition, the model also includes a compressing back plate made of acrylic glass and two spacers ensuring consistent compression of the soft plate. The geometry of all parts was chosen to be as close as possible to the clinical conditions (see Fig. 1b).

In experiments of removing the bacteria, the hard plate was made of an optical glass coated with a *Pseudomonas aeruginosa* monolayer attached to the glass surface as described below. A soft plate was molded in a 3D-printed template using fused deposition modeling printing technique (printer Prusa MK3). This process introduced a rough surface *Ra* = 8 µm [25], mimicking the irregularities found in periodontal and *peri*-implant pockets [26]. For comparison, the roughness of natural tooth surfaces ranges from Ra = 0.5–2 µm on enamel and up to Ra = 8 µm on root surfaces [27]. This setup allowed for precise quantification of bacteria removing efficiency under various Er:YAG laser treatment parameters, reflecting clinically relevant conditions.

For high-speed camera imaging of cavitation and fluid dynamics, the same zero-gap model was used as in experiments of removing the bacteria, however, additional surface refinements were made to explore the surface effects. In addition to rough-soft plate, we also fabricated a much smoother soft plate surface (*Ra* = 0.1 µm), molded into a polished aluminum template, which also minimized optical distortions and enhanced the clarity of cavitation bubble visualization. The coupled hard plate in these experiments was alternately devoid of bacteria or coated with them. Cavitation and fluid dynamics were systematically studied across the following four configurations:

a) a smooth-soft plate coupled with a bacteria-free hard plate (smooth/free),
b) a smooth-soft plate coupled with a bacteria-coated hard plate (smooth/coated),
c) a rough-soft plate coupled with a bacteria-free hard plate (rough/free) and
d) a rough-soft plate coupled with a bacteria-coated hard plate (rough/coated).

All soft tissue samples (Soft plates) were prepared under identical conditions and using the same formulations. The only variable changed was the roughness of the mold walls into which they were cast. No additional surface modifications (e.g., etching, plasma treatment, or mechanical polishing) were applied after curing.

The use of PDMS as the soft component and optical glass as the hard component in the zero-gap model was motivated by their suitability for controlled, reproducible, and optically accessible experiments. PDMS offers tunable mechanical properties that allow simulation of various soft tissue stiffness levels relevant to periodontal environments, while its optical transparency enables undistorted high-speed imaging of cavitation and fluid flow within the confined geometry. Optical glass provides a smooth, inert, and transparent surface, ideal for uniform bacterial monolayer deposition and consistent visualization of bacterial removal under microscopy. Although these materials do not replicate the exact surface chemistry or acoustic properties of clinical implants such as titanium, zirconia, or collagen-based scaffolds, their optical and mechanical stability was essential for the systematic analysis of laser-induced photoacoustic phenomena.

### 2.1 Growth and preparation of bacterial monolayers

Overnight culture (37 °C, 200 rpm) of *Pseudomonas aeruginosa* ATCC 27853 grown in 10 ml Tryptic Soy Broth (TSB) was centrifuged for 5 min at 4000 RCF. Supernatant was removed and pellet was washed 3-times in dH_2_O. After the third supernatant removal, the bacterial pellet was resuspended in 100 µl dH_2_O. This suspension was then used to form an attached monolayer of bacteria on the glass surface.

To prepare the bacterial monolayer we followed the methodology described by Pandur et al. [28]. For cell attachment, glass plates (dimensions: 20 mm × 20 mm × 3 mm) were utilized. They were initially cleaned using a sonication bath with 96 % ethanol for 20 min. Subsequently, the surface was rinsed with deionized water and allowed to air dry. Additionally, the glass surfaces underwent plasma cleaning (Harrick plasma, USA) at high frequency settings for 60 s. Immediately after plasma cleaning, 50 µl of poly-L-lysine (PLL) solution, prepared at 0.1 % (w/v) in deionized water (Sigma-Aldrich, USA), was applied to the surface and incubated at room temperature for 10 min. Next, the surfaces were rinsed with deionized water and left to air dry before being stored in a dark, dry environment prior to the use in experiments.

For the preparation of bacterial monolayers, 60 µl of a washed and concentrated bacterial suspension was added to the PLL-coated slides, followed by incubation at room temperature for 5 min to facilitate cell adherence. Subsequently, the surfaces were rinsed with deionized water to remove non-attached cells. The bacterial monolayer was then stained using a 1 % (v/v) crystal violet solution (Merck, Germany), with an incubation period of 2 min at room temperature. The excess stain was thoroughly washed with deionized water before proceeding with further analyses.

### 2.2 Removal of the bacteria

Glass plates with stained bacterial monolayer were inserted into a zero-gap model system and immersed 5 mm into the water bath (400 ml glass beaker with 3D printed plastic frame to hold the model in place), which ensured that the pocket was constantly filled with the distilled water. A pulsed Er:YAG laser system was used in this experiment (LightWalker AT-S, Fotona d.o.o., Slovenia). Through an articulated arm and handpiece (H14, Fotona, Slovenia), optical fiber tip of a diameter of 400 µm and length of 9 mm (FlatSWEEPS 400/9, Fotona, Slovenia) was inserted into the zero-gap model. The fiber tip insertion depth up to 3 mm was selected to represent clinically relevant shallow to moderate periodontal/*peri*-implant pocket depths, typically ranging from 3–5 mm in early to moderate periodontitis [1]. The fiber tip model and size was chosen for its compatibility with the laser system and ability to deliver the stated pulse energies, generating sufficient pressure waves for bacterial removal. Previous studies suggest that smaller-diameter fibers (e.g., 200–300 µm) increase energy density and cavitation efficiency due to a smaller focal volume [8], potentially enhancing distant-field (DF) cleaning. However, narrower fibers pose risks of mechanical tissue damage (e.g., wounding or scarring) and fiber breakage in confined pocket geometries.

To quantify bacterial removal, glass plates with a stained *Pseudomonas aeruginosa* monolayer were analyzed before and after Er:YAG laser treatment using an inverted microscope (Axio Observer 7, ZEISS, Germany) at 63x magnification. The resulting images were then analyzed using ImageJ software (v1.54 m). Initially, the gamma value was adjusted to 2 to correct for uneven intensity distribution, enhancing the visibility of faint bacteria while maintaining the intensity of brighter bacteria. Image segmentation was performed using the Otsu threshold method. To further refine cell separation, the watershed function was applied to resolve clumped cells. Cell counting was conducted with the “Analyze Particles” function, setting the size range from 0 to 300 µm.

For statistical analysis, each image was subdivided into four equal sized quadrants, and bacterial quantification was performed independently for each quadrant to account for spatial heterogeneity. The mean and standard deviation of bacteria counts across the four subregions were calculated and used to represent the variability within each image.

### 2.3 Acquisition and analysis of high-speed videos

High-speed imaging of cavitation dynamics within the zero-gap pocket was done by using a high-speed camera (Photron FASTCAM SA-Z) operating at 100,000 frames per second at reduced resolution 128x256 pix and a shutter speed of 248 ns. The camera was equipped with 1:1 macro lens (Sigma APO Macro, focal length 180 mm, aperture F2.8) providing resolution of 20 µm. The camera was positioned laterally to the zero-gap model, to capture the development, collapse, and propagation of cavitation dynamics during the Er:YAG laser treatment. A sequence of 100 frames was recorded after each laser pulse with a temporal resolution of 10 µs.

To ensure precise temporal synchronization between Er:YAG laser pulses and cavitation events, the camera was triggered by photodetector (Fotona, InAs Ser. No. 14000003) to detect the 2940 nm laser light. The photodetector’s analog signal was converted to a TTL (Transistor-Transistor-Logic) signal using a digital oscilloscope (Rigol, MSO1104Z), which was further connected to the camera’s external trigger input, initiating image acquisition simultaneously with the laser pulse.

**Fig. 2:**
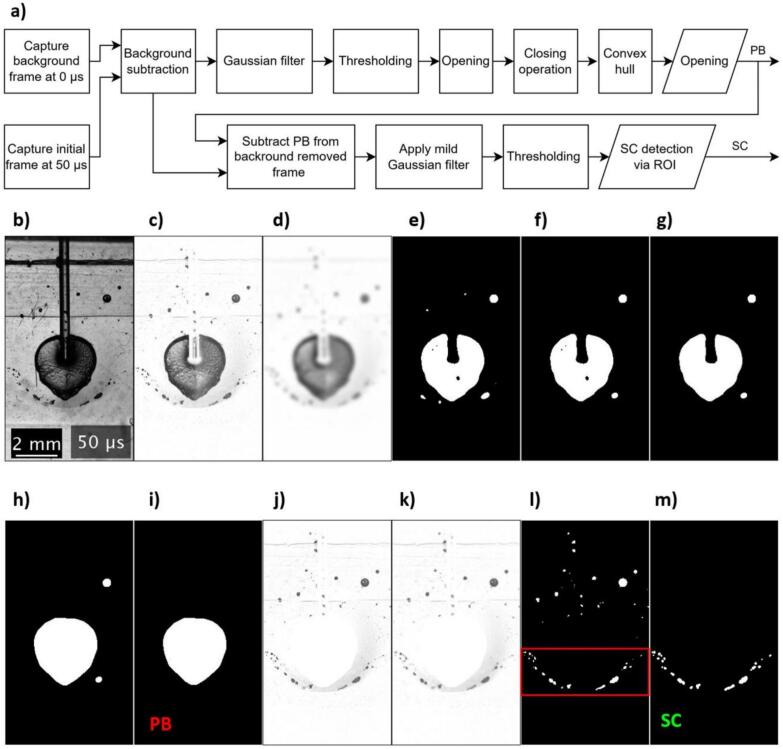
Image processing algorithm (a) for detection of cavitation dynamics and example of acquired image at various processing phases. b) Initial processing frame captured at 50 µs after the onset of the laser pulse. c) Background subtraction performed using a frame captured at 0 µs to isolate dynamic features related to the laser interaction. d) Application of a Gaussian filter to smooth the image and reduce noise, enhancing feature visibility. e) Thresholding applied to convert the smoothed image into a binary format for subsequent identification of features. f) Morphological opening operation executed to remove noise and irregularities. g) Closing operation conducted to fill in small holes from noise and irregularities. h) Convex hull algorithm utilized to close the upper gap in the primary bubble (PB), introduced by the FT. i) End result for PB detection using stronger opening to eliminate remaining larger anomalies. j) Subtraction of the detected PB area from the background-subtracted image (step b) to isolate features for secondary cavitation (SC). k) Mild Gaussian filtering. l) Thresholding applied to allow subsequent recognition. Region of interest (ROI) highlighted by a red rectangle. m) Final detection of SC achieved through selective ROI application, with the targeted area previously marked in step l.

The high-speed video data was processed utilizing a custom-developed algorithm to quantify the PB and SC area, as shown in Fig. 2.

Flow chart in Fig. 2a presents entire image processing algorithm, where the initial stage of the image processing takes a frame at the onset of the laser pulse (0 µs), which was used to remove the background from the subsequent frames in order to enhance visibility and isolate the cavitation dynamics. Further processing consisted of two stages. In the first stage we focused on detecting the PB area (Fig. 2c–i), beginning with noise reduction by employing Gaussian filtering with a kernel size *sigma* = 3 pix (Fig. 2d). For enhancing feature identification, thresholding was applied at a value of 75 % of maximal brightness, converting the image to a binary format (Fig. 2e). To refine the binary image several noise reduction and irregularities removal steps were undertaken, starting with a morphological opening using disk-shaped structural element of a 5-pixel radius eliminating small white noise spots (Fig. 2f). Subsequently, a closing operation using the same structural element was applied to fill in small gaps (Fig. 2g). To address irregularity introduced by the FT, a convex hull algorithm was utilized sealing the discontinuity around the upper region of the PB (Fig. 2h). As the final step, an additional opening operation was performed using a larger disk-shaped structural element with a 10-pixel radius, specifically aimed at removing more significant surrounding white anomalies, thereby finalizing accurate identification of the PB area (Fig. 2i).

In the second stage we addressed the detection of the SC area by employing similar techniques, starting with the already existing background-subtracted image (Fig. 2c), from which the area corresponding to the PB was excluded to focus on the remaining features (Fig. 2j). A finer Gaussian filter with a kernel size *sigma* = 0.7 pix was applied to subtly smooth the remaining details (Fig. 2k), setting the stage for less noisy thresholding result, which was applied with a threshold value of 76 % (Fig. 2l). Following this, a region of interest (ROI) was defined to remove irrelevant areas. The final detection of SC (Fig. 2m) was achieved by applying the binary mask from the ROI defined as a horizontal band extending from the height of the FT downward by 2.3 mm (Fig. 2l), while areas outside this band contained only residual artifacts that escaped previous filtering steps.

**Fig. 3:**
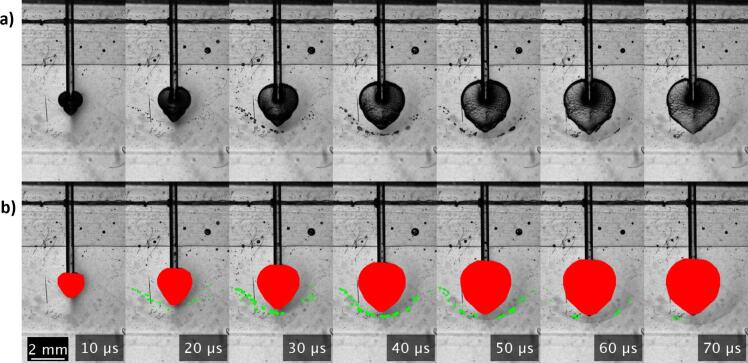
Image processing for detection of cavitation dynamics over time on smooth/free configuration. a) Sequence of initial frames captured at 10 µs intervals from 10 µs to 70 µs after the onset of the laser pulse. b) The same sequence of frames annotated to highlight the detected cavitation features: PB are marked in red and SC events are indicated in green. Each frame utilizes the image processing algorithm described in Fig. 2.

All parameters used in the image processing steps were selected through empirical testing, aiming to isolate the desired features, specifically the PB and SC areas, consistently across multiple recordings. To further illustrate the quantization of cavitation dynamics over time, the image processing algorithm described earlier is demonstrated in Fig. 3 across a series of frames captured at 10 µs intervals. This sequential image processing showcases the progression of cavitation events from 10 µs to 70 µs following the laser pulse onset. The PB (red) and SC (green) events are distinctly marked, providing a clear visualization of the temporal development of cavitation phenomena in the zero-gap region of the model system.

Utilizing the developed algorithm for PB and SC detection, the areas of PB (A_PB_) and SC (A_SC_) were quantified by counting the pixels within each designated area and then converting the values into actual surface areas in square millimeters using a calibration scale. In the following results, unless presented as a function of time, A_PB_ and A_SC_ refer to the average of the maximum areas observed across multiple cavitation events induced by several laser pulses (N > 30) of the same parameters.

Visual analysis of high-speed video frames revealed displacement of individual visible particles in the distant field (DF) region. By comparing their positions across consecutive frames acquired at 10 µs intervals, the apparent displacement velocity was estimated based on frame-to-frame movement, providing an indication of local fluid motion.

### 2.4 Laser parameters and modalities

The 2940 nm wavelength emitted by the Er:YAG laser was selected due to its strong absorption in water, enabling efficient and localized vapor bubble formation essential for cavitation-driven photoacoustic effects. Its proven clinical safety profile in dental procedures further supports its suitability for this application.

Two laser modalities were used during the experiments, Ultra-Short Pulse (USP) and ASW. USP was the baseline modality, delivering single pulses of energy set at 10 mJ, 20 mJ, or 30 mJ, with a constant frequency of 15 Hz. This modality was employed to generate PB, providing controlled and consistent bubble dynamics for bacteria removal. The treatment time for both modalities was 10 s.

ASW, on the other hand, consists of two successive USP pulses, each having the same energy setting as the single USP pulse (e.g., 10 mJ per pulse for a total energy delivery of 20 mJ per pair). This dual-pulse modality enhances cavitation dynamics by employing a continuously sweeping delay between both pulses back and forth in increments of 10 microseconds in the range from 200 to 650 microseconds to target the resonance conditions. When the resonance is achieved, the cavitation dynamics are amplified [29], enabling more efficient removal of the bacteria compared to the single-pulse modalities.

The selected range of laser parameters and soft plate stiffness levels was based on clinically relevant conditions. However, we intentionally explored a broader range of pulse energies, dual-pulse delays, and fiber tip positions to systematically investigate their individual and combined effects on cavitation dynamics and bacterial removal. This parametric exploration provides a foundation for future optimization under specific clinical constraints.

## 3 Results

**Fig. 4:**
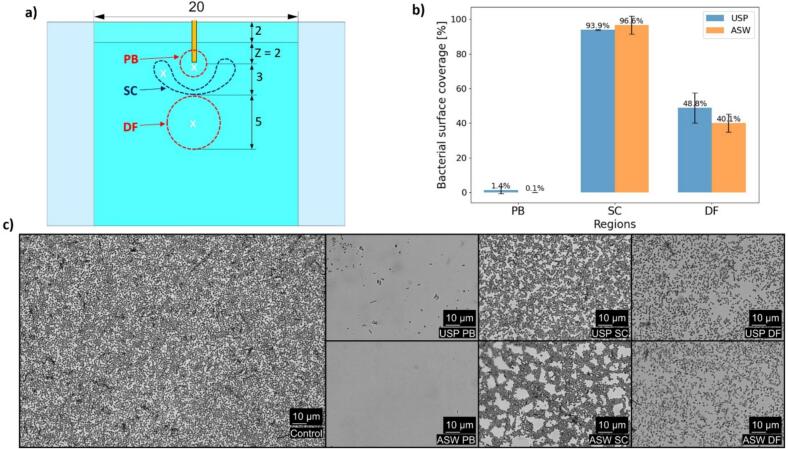
Removal of the bacteria from the zero-gap model during Er:YAG laser photoacoustic treatment in the rough/coated configuration. All treatments shown were performed at 20 mJ, 15 Hz, and 10 s treatment time. a) Frontal view of the zero-gap model (corresponds to the cross-section illustrated in Fig. 1b) showing the insertion depth of the FT and the key areas: primary bubble (PB), secondary cavitation (SC), and a distant field (DF) where cleaning effect was also observed but could not be explained with PB and SC effects. Locations marked with ‘x’ indicate the specific positions within the pocket where bacterial coverage was analyzed using microscopy. b) Bar graph presenting the fraction of remaining bacteria relative to the control across different regions for two modalities: Ultra-Short Pulse (USP, blue) and Auto-SWEEPS (ASW, orange). Each bar represents the mean of N = 4 measurements within image. c) Microscopic images showing individual P. aeruginosa bacterial cells (dots) in a monolayer attached to the glass surface in control (prior to the laser treatment) and in different areas around the fiber tip after the laser treatment for PB, SC, and the DF areas, for both USP (upper row) and ASW (lower row) modalities are given.

To evaluate the efficiency of Er:YAG laser bacteria removal and its dependence on various parameters, a series of experiments were conducted using the zero-gap model, the results of which are shown in Fig. 4.

The frontal view of the model (Fig. 4a) highlights three distinct regions of interest: the PB region, the SC region, and DF, where cleaning effects were observed but could not be as easily explained by primary and secondary cavitation bubbles. As expected, the results demonstrate high removal efficiencies in the PB region where more than 98 and 99.9 % of bacteria were removed with USP and ASW laser modalities, respectively. These values are shown in the bar graph (Fig. 4b), which presents the mean bacterial removal for each region based on quadrant-wise analysis. The corresponding cleaning efficiency was much lower in the region dominated by secondary cavitation bubbles. Microscopic images (Fig. 4c) serve as the foundation for these findings, showing remaining bacteria patterns across PB and SC regions for both laser modalities. The distribution of bacterial cells on the surface was disturbed by USP and ASW laser modalities. However, the bacterial surface coverage decreased only moderately relative to the control sample, suggesting that secondary cavitation mainly redistributed bacterial density on the surface. The redistribution of bacterial cells was much more pronounced for the ASW laser modality. Surprisingly, cleaning was observed in the DF region where no secondary bubbles were observed. Here both surface coverage and redistribution of bacterial cells on the surface were observed.

These results confirm the efficacy of Er:YAG laser photoacoustic bacteria removal in the PB and SC regions while raising questions about the mechanisms driving the observed cleaning effects in the DF region. The following sections explore these mechanisms in greater detail, beginning with the impact of laser parameters on bacteria removal.

### 3.1 Laser fiber tip depth

**Fig. 5:**
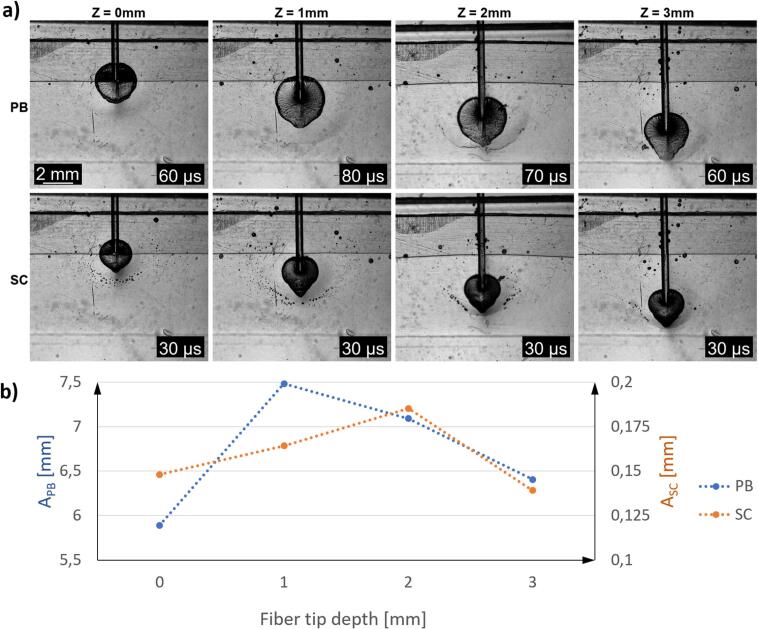
Effect of FT depth on cavitation bubble dynamics in the smooth/free configuration of the zero-gap model during laser treatment utilizing USP modality (20 mJ, 15 Hz). a) Visualization of primary bubbles (top row) and SC (bottom row) at four FT depths. Frames demonstrate the largest primary bubbles and SC events, along with their occurrence times. b) Graphical representation of average cavitation size observed. The sizes of the primary cavitation bubbles (A_PB_, left vertical axis) and SC bubbles (A_SC_, right vertical axis) are plotted as a function of depth positions along the Z-axis, ranging from 0 mm to 3 mm.

Since the most efficient cleaning was observed in PB region, we studied if the position of FT influences cavitation dynamics in the model system. High-speed imaging revealed that the center of the PB and SC follows the movement of the FT (see Fig. 5a). The sizes of both PB and SC (see Fig. 5b) increased when the FT was moved from a depth of 0 mm to 1 mm. This increase can be attributed to the FT transitioning from the sloped wedge region of the soft plate into the constrained zero-gap pocket. At 1 mm depth, the cavitation bubbles were compressed laterally, resulting in a larger surface area compared to bubbles formed at shallower depths.

At greater depths, such as 2 mm and 3 mm, the cavitation bubbles become smaller. This reduction in size occurs because deeper insertion of FT requires displacement of a larger mass of soft material, which imposes additional constraints on bubble expansion. The observed variations in cavitation bubble size and shape highlight the importance of FT positioning in optimizing cavitation dynamics for the removal of the bacteria within constrained geometries.

### 3.2 Cavitation evolution employing dual laser-pulses

**Fig. 6:**
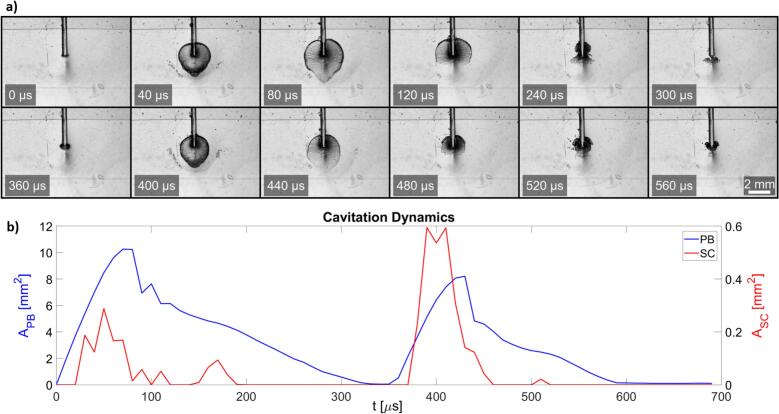
Typical development of PB and SC during dual-pulse AutoSWEEPS modality in the smooth/free configuration of the zero-gap model. a) High-speed camera images showing the evolution of cavitation bubbles during the first (0–300 µs) and the second pulse (360–560 µs). SC is distinctly observed at 40 µs and 400 µs frames. b) Line graph showing area of primary bubble (A_PB_) and secondary cavitation (A_SC_) over time corresponding to growth and collapse events observed in a).

To further investigate the mechanisms enabling the removal of the bacteria, we measured the development of cavitation dynamics during the dual-pulse ASW modality. High-speed imaging captured the evolution of resonance cavitation events, as shown in Fig. 6a.

The first row of images shows the growth and collapse of the primary cavitation bubble during the first pulse (0–300 µs), while the second row demonstrates the cavitation dynamics of the second pulse (360–560 µs). Distinct SC events are clearly observed at 40 µs and 400 µs, correlating with expansion stages of primary bubbles.

Quantitative analysis of the high-speed videos was performed, detecting the area of the PB and SC. Fig. 6b shows the temporal progression of the bubble surface area during both pulses, revealing a two-phase collapse. The initial expansion is followed by a rapid decrease in bubble size, followed by a slower collapse up to 350 µs. This observation can be explained by the rapid closure of the lower portion of the bubble which can be observed from 80 µs to 120 µs during the first pulse, while the upper region persists for a longer duration, as evident in the high-speed images (e.g., Fig. 6a). A similar effect is observed with the second primary bubble at approximately 440 µs. Interestingly, the primary bubble generated by the second pulse is smaller and of shorter duration than the first, yet it induces a larger SC, which can potentially contribute to better removal of the bacteria in surrounding regions as experimentally observed.

### 3.3 Role of soft plate stiffness, energy and laser modality

**Fig. 7:**
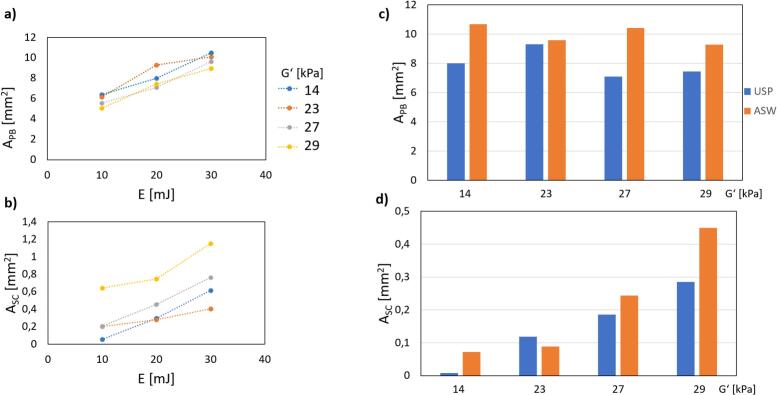
The impact of soft plate stiffness, energy and laser pulse modality on cavitation dynamics in the smooth/free configuration of the zero-gap model. a), b) The areas of primary (A_PB_) and secondary (A_SC_) cavitation bubbles were evaluated as functions of laser energy at four soft plate stiffness levels in terms of storage modulus (G') using the Ultra-Short Pulse (USP) modality. c), d) Comparisons between USP and the first pulse of Auto-SWEEPS (ASW) across the different stiffness levels. All experiments employed pulses of 20 mJ energy at 15 Hz.

By using both laser modalities (USP and ASW), we investigated also the effects of soft plate stiffness (expressed as storage modulus *G*') and laser pulse energy on cavitation dynamics. The results indicate that both PB and SC sizes increase with higher laser energy (Fig. 7a, b). Interestingly, comparisons between USP and the first pulse of the ASW modality (Fig. 7c, d) revealed that ASW on average produced larger PB as well as SC, even when considering only its first pulse. This effect is likely due to the additional energy input in the dual-pulse configuration, which alters the thermal and dynamic parameters of the system. Namely, by increasing the temperature and consequently reducing the cavitation threshold.

The soft plate stiffness had the opposite effect on the size of the primary compared to the secondary bubbles. As shown in Fig. 7b, d, stiffer soft plate resulted in significantly larger SC, but smaller PB (Fig. 7a, c), highlighting the importance of material stiffness in modulating cavitation dynamics. These findings provide deeper insights into the relationship between material properties, energy, and laser modality in optimizing removal of the bacteria.

### 3.4 Role of the second pulse delay in enhancing secondary cavitation

**Fig. 8:**
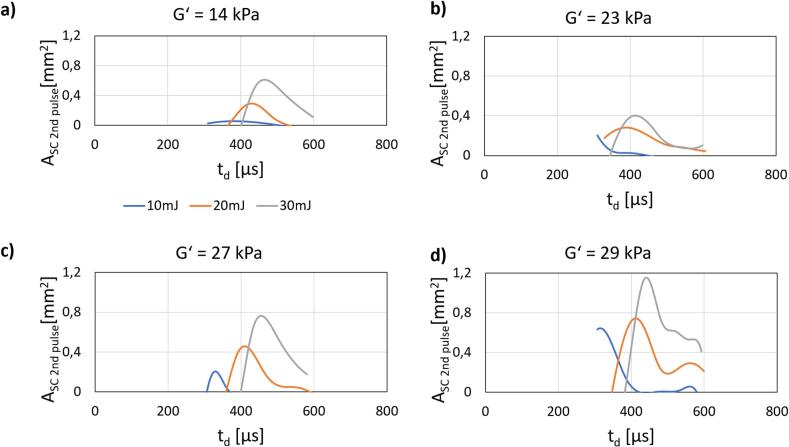
Enhancement of SC following the second pulse in ASW modality in the smooth/free configuration of the zero-gap model. This Fig. presents a series of four graphs, each showing the variation in the maximum area achieved by SC (A_SC 2nd pulse_) as a function of the delay between pulses (t_d_) and the stiffness of the soft plate. The graphs are labeled a) to d), corresponding to soft plate stiffness in terms of storage modulus (G').

To further explore the mechanisms of bacteria removal in regions beyond the observable cavitation, we investigated the influence of the delay between pulses in the ASW modality across different soft plate stiffness levels (Fig. 8). The results indicate that, for all tested stiffnesses, there exists an optimal delay between the pulses that maximizes SC. Achieving this optimal delay, however, is challenging to predict, as it is highly dependent on the surrounding geometry and material properties, which introduces variability into the resonance conditions. Notably, less stiff material exhibited significantly less SC compared to stiffer ones. This is particularly important in clinical context, as softer material represents more inflamed gingival tissue, where cleaning is the most critical. Here increasing the laser energy significantly increases SC. These findings highlight the complexity of optimizing pulse timing to enhance SC under clinically relevant conditions.

### 3.5 Exploration of surface roughness effects

**Fig. 9:**
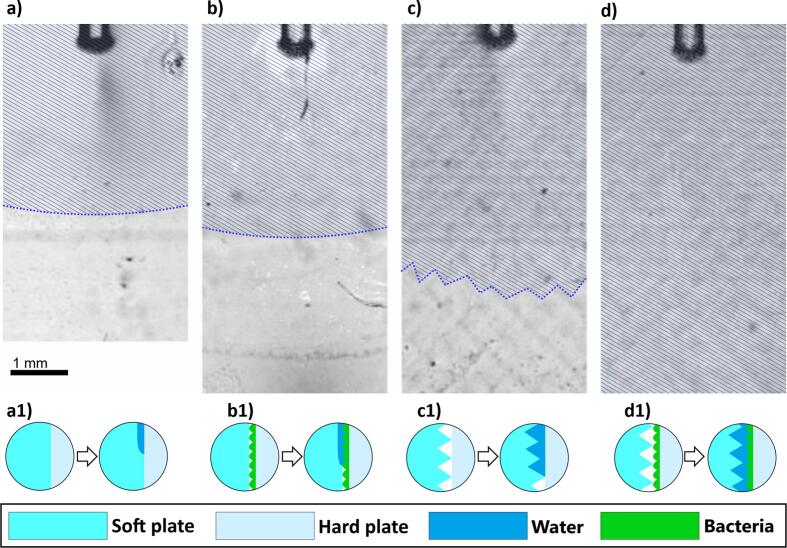
Influence of surface roughness on water penetration and bacteria removal during Er:YAG laser photoacoustic treatment using USP modality. (a-d) High-speed camera images illustrating water wave penetration under four configurations of surface conditions between a soft and hard plate: a) a smooth soft plate coupled with bacteria-free hard plate (smooth/free), b) a smooth soft plate coupled with a bacteria-coated hard plate (smooth/coated), c) a rough soft plate coupled with a bacteria-free hard plate (rough/free), d) a rough soft plate coupled with bacteria-coated hard plate (rough/coated). Water penetration is visualized with blue hatching, indicating the extent of fluid penetration for each condition. (a1-d1) Schematic depiction of surface conditions and their effect on water penetration during the laser treatment. Each case includes two depictions: one highlighting the surface conditions before the laser treatment, and the other showing the extent of water penetration after laser treatment.

Surface properties emerged as a critical factor influencing water penetration and bacteria removal, as shown in Fig. 9.

The results clearly demonstrate that rougher and bacteria coated surfaces enhance water penetration, which enables SC occurrence even in DF region (see video 1 in the supporting material), therefore also providing the cleaning effect in DF observed in Fig. 4c. Water penetrates least in smooth/free surface configuration. In smooth/coated configuration it penetrates deeper. In the rough/coated configuration, water penetration was so extensive that its limit could not be observed in the model system. These findings emphasize the significant role of surface roughness on fluid dynamics and bacteria disruption during Er:YAG laser photoacoustic treatment.

Careful high-speed video analysis reveals that the cavitation-related phenomena induces water flow reaching up to 10 m/s in the DF. In addition, microscopic secondary cavitation events are also observed (see d in Video 1 in the supporting material), further enhancing the removal of the bacteria. The rapid flow contributes to bacteria removal by causing deeper water penetration, creating the water pathway for pressure waves, while the high speed also enhances the pressure waves leading to SC occurrence. Additionally, the flow supports a rinsing mechanism, emphasizing the combined role of surface roughness, secondary cavitation, and rinsing in facilitating effective cleaning during Er:YAG laser photoacoustic treatment.

## 4 Discussion

This study advances the understanding of the mechanisms involved in Er:YAG laser-induced photoacoustic bacteria removal in constrained geometries, such as periodontal and *peri*-implant pockets. The findings reveal several critical factors influencing the cleaning effect, including cavitation dynamics, surface roughness, and laser parameters.

The most intriguing observation of this study is the removal of the bacteria in DF region, where neither cavitation nor fluid penetration was observed on the high-speed camera. Since these two experiments were performed separately it raises the question of whether all conditions were truly identical in both cases. Unable to identify any obvious differences, we systematically examined important cavitation parameters in depth, initially focusing on FT positioning, laser energy, pulse modality, and soft plate stiffness. These parameters influenced cavitation dynamics and bacterial removal, particularly in PB and SC regions, consistent with previous studies investigating laser-induced fluid motion in constrained and zero-gap geometry [11,14]. Notably, deeper FT insertion improved the cleaning effect by shifting cavitation and fluid motion deeper within the constrained space. Moreover, dual-pulse modalities, particularly ASW, enhanced bacterial removal by generating more SC. However, optimizing these parameters for inflamed tissues, as represented by a softer plate, remains a challenge that requires further investigation.

Despite these efforts, no combination of parameters could fully account for the observed cleaning effect in the DF region. This discrepancy led us to identify surface roughness conditions as a previously overlooked critical parameter. To test this, we conducted the high-speed camera experiment while systematically testing four interface configurations: the soft plate was either smooth or rough, while the hard plate was either coated with bacteria or left uncoated. Interestingly, both a rough-soft plate and a bacteria-coated hard plate facilitated deeper water penetration, ultimately leading to the occurrence of secondary cavitation deeper in the zero- gap. When only the hard plate was coated with bacteria, penetration depth increased by 10 %, whereas a rough-soft plate alone resulted in a 50 % increase. The combination of both effects further amplified penetration, increasing it by more than 100 %, extending beyond the visible field of observation.

The particles observed in video 1 were most likely not detached bacteria, but rather impurities present in the fluid, moving around during photoacoustically-induced streaming. Nevertheless, their motion is representative of how bacteria are displaced and redistributed under these conditions, highlighting the critical role of surface roughness not only in enabling cavitation but also in enhancing fluid dynamics and rinsing effects (measured velocity of fluid in DF is 10 m/s), emphasizing the importance of accurately replicating the texture and geometry of periodontal and *peri*-implant pockets in experimental models. Based on micrographs, bacteria were both displaced from and redistributed.

These findings suggest that surface irregularities significantly alter fluid behavior in the zero-gap model, thereby enhancing bacterial removal even in regions distant from the laser fiber tip. Previous studies have demonstrated that surface roughness modulates wettability, either enhancing hydrophilicity or hydrophobicity, depending on morphology and surface chemistry [15]. In our zero-gap model, the rough soft plate (Ra = 8 µm) and bacterial monolayer likely reduce the contact angle on the hydrophilic PDMS surface, enhancing wettability compared to the smooth configuration (Ra = 0.1 µm) [15,16]. Such variations influence how water penetrates and interacts with textured surfaces, promoting deeper infiltration into narrow geometries. In addition, bacterial biofilms contribute to micro-scale roughness, further altering surface energy and local fluid dynamics [16]. Beyond fluid transport, surface roughness promotes cavitation by providing nucleation sites. Micro-crevices and asperities can entrap gas, lowering the local pressure threshold for cavitation [18]. Analytical and experimental studies have shown that sharp-edged topographies create localized low-pressure zones, which facilitate vapor bubble formation and collapse [17].

Recent imaging and theoretical studies support these observations. Eskin et al., using ultrafast X-ray and high-speed optical imaging, showed that rough fluid–solid interfaces amplify local pressure peaks and enhance cavitation activity through scattering and confinement effects [12,13]. These findings align with crevice nucleation theory, reinforcing that rough surfaces promote cavitation at lower pressure amplitudes [30]. In addition, high-speed imaging of cavitation near textured surfaces reveals micro-jet formation and acoustic microstreaming, both of which can disrupt biofilms even in regions not directly reached by cavitation bubbles [31]. These effects are strengthened by constructive interference and enhanced energy deposition, as demonstrated in cavitation-based cleaning performance on structured substrates [6].

To model these mechanisms more quantitatively, fluid–structure interaction (FSI) simulations offer a promising approach. FSI studies by Kojima and Inaba have shown that variations in surface wettability and stiffness alter wave transmission and pressure localization at soft–solid interfaces [32]. Additionally, simplified FSI models of bubble collapse near compliant boundaries reveal that wall elasticity dampens peak pressures by approximately 10 %, emphasizing the importance of accounting for soft tissue mechanics when modeling DF effects [33].

These shows that surface roughness plays a central role in shaping wave propagation, cavitation behavior, and fluid streaming in narrow periodontal geometries. Future research should pursue high-fidelity FSI and multiphysics simulations that incorporate clinically realistic textures and mechanical properties to guide optimization of distant-field laser-assisted cleaning strategies.

Although reactive oxygen species (ROS) were not measured in this study in photochemical systesm such as photoacoustic treatments in the presence of photosensitizers or organic solvents may play a role in generating singlet oxygen (^1^O_2_) or superoxide reactive species. Degassing these systems should in principle suppresses photoinduced ROS formation. Nevertheless, we have previously shown that the main effect of Er:YAG treatment in non-degassed samples is not the destruction of bacterial cells but rather their removal from the model pocket through mechanical shear forces [14].

Although from the biological point of view 50 to 60 % reduction in bacterial surface coverage may not seem a lot, as bacteria may reach the same surface density during one generation, the results are nevertheless significant as there are no other methods that are applicable in such narrow spaces for removal of the bacteria and can reduce inflammation pressure. The cleaning efficiency can be further improved by increasing the laser energy or more importantly by implementing double pulse laser modality, as demonstrated in Fig. 4. This approach reduces the remaining bacterial presence from 48 % with USP to 40 % with ASW, achieving an additional 8 % removal.

Importantly, unlike direct laser ablation techniques, the approach presented here relies on indirect photoacoustic action. The Er:YAG laser energy is fully absorbed in the irrigant, generating vapor bubbles that induce cavitation and fluid motion without direct interaction with implant surfaces. As a result, this method is inherently material-independent and non-destructive, enabling its safe application across a variety of implant types, including titanium, zirconia, and enamel, without altering their surface properties. Nevertheless, material-specific optimization of treatment parameters may still be required to account for differences in geometry, wettability, and acoustic response.

The findings emphasize the need for advanced experimental models that better mimic clinical conditions, including tissue stiffness, constrained geometries, and surface roughness, as demonstrated in this study. Such realistic models are essential not only for optimizing treatment parameters but also for understanding the underlying mechanisms of laser-induced photoacoustic cleaning. Future research should focus on refining these models to better replicate inflamed and structurally compromised tissues, and on exploring the complex interplay between laser parameters, cavitation dynamics, and fluid motion.

Surface modifications or interfacial enhancements of implant materials could significantly influence the outcomes of Er:YAG laser-induced photoacoustic bacterial removal observed in our study. The zero-gap model demonstrated that increased surface roughness (Ra = 8 µm, rough soft plate) enhances water penetration and cavitation dynamics, improving bacterial removal in primary bubble (PB), secondary cavitation (SC), and distant-field (DF) regions (Fig. 4, Fig. 9). Clinical implant materials, such as titanium, typically exhibit roughness values of Ra = 1–5 µm [26], which could similarly promote cavitation by providing nucleation sites, potentially amplifying cleaning efficacy as seen in our rough/coated configuration. However, advanced surface modifications, such as nano-texturing, may alter wettability and reduce water penetration, limiting photoacoustic effects [16].

However, several potential challenges must be addressed to enable successful clinical translation of this technology. Anatomical variability in periodontal and *peri*-implant pockets, such as differences in geometry, compliance, and inflammation, can affect water retention and cavitation behavior. Maintaining a stable fluid environment in vivo, as achieved in vitro, is technically demanding and may impact treatment reproducibility. Furthermore, the efficacy of distant-field cleaning depends strongly on precise fiber tip positioning and controlled energy delivery. In a clinical setting, limited access, patient movement, and lack of visual guidance may reduce the consistency of cavitation-mediated effects. Surface roughness, hydration, and tissue heterogeneity also contribute to unpredictable cavitation thresholds, increasing the risk of suboptimal cleaning or unintended tissue effects.

Er:YAG laser-induced photoacoustic cleaning has shown promising non-invasive potential for disrupting biofilms, effectively reaching and decontaminating anatomically challenging areas through photoacoustic mechanisms [34]. Therefore, the laser device and fiber tip used in this research have direct clinical application in the non-surgical treatment of periodontal disease, particularly in anatomically constrained/zero-gap pocket conditions where conventional mechanical debridement is limited. Drawing inspiration from related biomedical technologies, such as the cavitation catheter used in dual-frequency ultrasound-assisted thrombolysis for deep vein thrombosis [35] and the cavitation heart pump designed for microbubble-enhanced sonothrombolysis in ventricular assist devices [36], future device development could explore application-specific design modifications that intentionally modulate cavitation dynamics to enhance clinical efficacy.

While the in vitro model isolates the photoacoustic mechanism, the selected Er:YAG laser parameters (10–30 mJ, 15 Hz) are well within clinically accepted safety limits. The laser fluences at the fiber tip used in this study ranged from approximately 8  J/cm^2^ to 24  J/cm^2^ per pulse, delivered through a 400 µm fiber. These fluence values are consistent with previously reported ranges for Er:YAG laser-induced photoacoustic cleaning in dental applications [37]. Since the laser energy is absorbed by water and not directly by tissue, thermal damage risk is minimal. Measurements presented in the Supplementary Material (Fig. S1) indicate temperature increase of approximately 3 K after 10 s of procedure. Prolonged exposure (60 s) results in a temperature rise of less than 5 K. Notably, the fiber tip was stationary during these measurements, leading to higher temperatures than expected under clinical conditions, where it is continuously moved.

The insertion and removal of the fiber tip into the periodontal or *peri*-implant pocket is not associated with discomfort, as the tip is small and smooth, and the procedure is comparable to standard periodontal probing. In photoacoustic treatment, there is no significant temperature elevation during this procedure, but we may expect slightly more vibrations due to cavitation effects. Local anesthesia will be sufficient to prevent any discomfort. Nonetheless, in vivo studies are necessary to confirm that tissue integrity is maintained under variable biological conditions.

Despite its strengths, the present study has several limitations. First, the in vitro zero-gap model simplifies biological conditions and does not capture the full mechanical and biochemical variability of human periodontal tissues. Second, fluid retention within the pocket is idealized, whereas maintaining stable water-filled conditions intraorally is more difficult. Third, the range of surface roughness tested was limited, and future work should explore a broader spectrum of clinically relevant textures. Lastly, while high-speed imaging provides indirect insight into cavitation and fluid flow, no direct pressure measurements were performed. Future studies should integrate pressure field measurement [38] or fiberoptic probing [39] to validate wave propagation and cavitation dynamics more rigorously.

## 5 Conclusions

This study provides new insights into the mechanisms of Er:YAG laser-induced photoacoustic removal of bacteria in constrained geometries with rough surfaces. It was observed that bacterial removal occurred in regions not in direct contact with the fiber tip, where cavitation had not been previously detected, suggesting the involvement of additional mechanisms such as enhanced pressure wave propagation and rinsing. The rough surfaces and bacteria-coated surfaces were found to promote significantly deeper water penetration and influenced cavitation dynamics, extending their non-contact effects during photoacoustic treatment. Additionally, deeper fiber tip insertion improved the cleaning effect by shifting cavitation and fluid motion deeper within the constrained space. Higher laser energies and the implementation of dual-pulse modality (ASW) further enhanced secondary cavitation formation, leading to increased bacterial removal. These findings emphasize the relevance of the realistic models that closely mimic clinical conditions to optimize Er:YAG laser-induced photoacoustic removal of bacteria in periodontal and *peri*-implant therapy, ultimately enabling precise optimization of treatment parameters to improve clinical outcomes.

To support clinical translation, future work should focus on (i) in vivo validation in relevant animal models, (ii) development of anatomically accurate soft tissue phantoms, (iii) integration of real-time cavitation sensing and adaptive laser control, and (iv) parameter optimization under clinical boundary conditions. These steps will enable rigorous safety assessment and protocol refinement, ultimately paving the way for clinical pilot studies in dental practice.

## Declaration of Generative AI and AI-assisted technologies in the writing process

During the preparation of this work the author(s) used Chat GPT in order to improve language and readability. After using this tool/service, the author(s) reviewed and edited the content as needed and take(s) full responsibility for the content of the publication.

## CRediT authorship contribution statement

**Dominik Šavli:** Writing – original draft, Visualization, Software, Investigation. **Marko Volk:** Writing – original draft, Visualization, Validation, Methodology, Investigation. **Katja Molan:** Writing – review & editing, Methodology. **Saša Terlep:** Writing – review & editing, Methodology. **Špela Levičnik-Höfferle:** Writing – review & editing, Methodology. **Aleš Babnik:** Writing – review & editing, Methodology, Investigation, Conceptualization. **Mojca Trost:** Writing – review & editing, Methodology, Conceptualization. **Boris Gašpirc:** Writing – review & editing, Investigation, Conceptualization. **Matjaž Lukač:** Writing – review & editing, Validation, Methodology. **David Stopar:** Writing – review & editing, Validation, Methodology, Investigation, Funding acquisition, Conceptualization. **Matija Jezeršek:** Writing – review & editing, Writing – original draft, Supervision, Methodology, Investigation, Funding acquisition.

## Declaration of competing interest

The authors declare that they have no known competing financial interests or personal relationships that could have appeared to influence the work reported in this paper.
