# Supplementary material for: The effect of surface roughness on the Er:YAG laser-induced photoacoustic removal of bacteria in zero-gap periodontal/peri-implant pocket model
Source: Ultrason Sonochem. 2025 Jul 7;120:107458. doi: 10.1016/j.ultsonch.2025.107458 (PMC12343481; doi:10.1016/j.ultsonch.2025.107458)
Supplement: Supplementary Data 1 [file mmc1.docx]

**Supplementary information**

**The effect of surface roughness on the Er:YAG laser-induced photoacoustic removal of bacteria in zero-gap periodontal/peri-implant pocket model**

Dominik Šavli^1^, Marko Volk^2^, Katja Molan^7^, Saša Terlep^3^, Špela Levičnik-Höfferle^3^, Aleš Babnik^1^, Mojca Trost^4^, Boris Gašpirc^4^, Matjaž Lukač^3,5,6^, David Stopar^2^, Matija Jezeršek^1*^

^1^ University of Ljubljana, Faculty of Mechanical Engineering, Aškerčeva cesta 6, 1000 Ljubljana.

^2^ University of Ljubljana, Biotechnical Faculty, Department of Microbiology, Večna pot 111, 1000 Ljubljana, Slovenia.

^3^ Fotona d.o.o., Stegne 7, 1000 Ljubljana, Slovenia.

^4^ University of Ljubljana, Medical Faculty, Department of Oral Medicine and Periodontology, Vrazov trg 2, 1000 Ljubljana.

^5^ Institut Jozef Stefan, Jamova 39, 1000 Ljubljana, Slovenia.

^6^ University of Ljubljana, Faculty of Mathematics and Physics, Jadranska 19, 1000 Ljubljana, Slovenia

^7^ University of Novo Mesto, Faculty of Health Sciences, Na Loko 2, 8000 Novo mesto

*Corresponding author:

Prof. dr. Matija Jezeršek

University of Ljubljana, Faculty of Mechanical Engineering, Aškerčeva cesta 6, 1000 Ljubljana, Slovenia

E-mail: matija.jezersek@fs.uni-lj.si

**Temperature measurement**

Temperature rise during laser irradiation was measured using a high-speed thermal camera (TELOPS Fast M3K, spectral sensitivity: 1.5–5.4 µm, resolution: 320 × 256 pixels, frame rate: 3100 FPS). For this purpose, the optical glass plate in the experimental setup was replaced with a 1 mm thick sapphire window (Edmund Optics) to ensure sufficient infrared transmittance up to 5.4 µm.

The same Er:YAG laser system (LightWalker AT-S, Fotona d.o.o., Slovenia) and optical fiber tip (FlatSWEEPS 400/9, 400 µm diameter) were used as in bacterial removal and cavitation monitoring experiments. The Ultra-Short Pulse (USP) modality was employed, delivering single laser pulses at 20 mJ energy with a repetition rate of 15 Hz. The total irradiation time was 60 seconds.


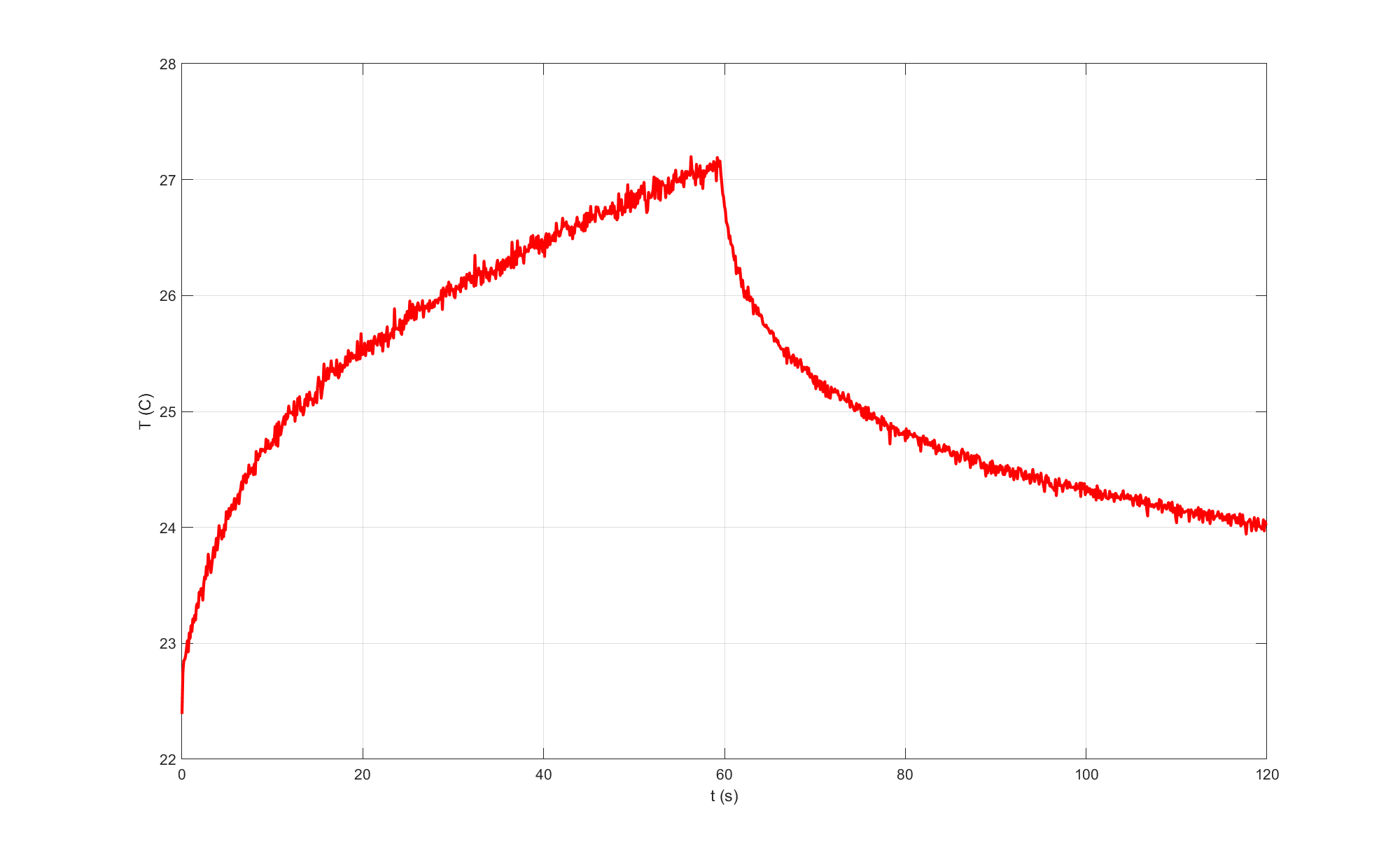


**Figure S1:** Temperature measurements during a laser treatment (USP modality, 20 mJ pulse energy @ 15 Hz).

Fig. S1 shows an average temperature in the region surrounding the fiber tip (within 1 mm radius), derived from thermal camera measurements. The measurements show that the region surrounding the fiber tip experienced an average temperature increase of approximately 3 K after 10 seconds of continuous irradiation. After 60 seconds, the temperature rise is 5 K.
